# Self-Reported Preferences for Help-Seeking and Barriers to Using Mental Health Supports Among Internal Medicine Residents: Exploratory Use of an Econometric Best-Worst Scaling Framework for Gathering Physician Wellness Preferences

**DOI:** 10.2196/28623

**Published:** 2021-10-06

**Authors:** Andrew Wu, Varsha Radhakrishnan, Elizabeth Targan, Timothy M Scarella, John Torous, Kevin P Hill

**Affiliations:** 1 Beth Israel Deaconess Medical Center Harvard Medical School Boston, MA United States

**Keywords:** residency program, choice, burnout, wellness

## Abstract

**Background:**

Burnout interventions are limited by low use. Understanding resident physician preferences for burnout interventions may increase utilization and improve the assessment of these interventions.

**Objective:**

This study aims to use an econometric best-worst scaling (BWS) framework to survey internal medicine resident physicians to establish help-seeking preferences for burnout and barriers to using wellness supports by quantifying selections for 7 wellness support options and 7 barriers.

**Methods:**

Internal medicine resident physicians at our institution completed an anonymous web-based BWS survey during the 2020-2021 academic year. This cross-sectional study was analyzed with multinomial logistic regression and latent class modeling to determine the relative rank ordering of factors for seeking support for burnout and barriers to using wellness supports. Analysis of variance with Tukey honest significant difference posthoc test was used to analyze differences in mean utility scores representing choice for barriers and support options.

**Results:**

Of the 163 invited residents, 77 (47.2% response rate) completed the survey. Top-ranking factors for seeking wellness supports included seeking informal peer support (best: 71%; worst: 0.6%) and support from friends and family (best: 70%; worst: 1.6%). Top-ranking barriers to seeking counseling included time (best: 75%; worst: 5%) and money (best: 35%; worst: 21%).

**Conclusions:**

Overall, our findings suggest that low utilization of formal mental health support is reflective of resident preferences to seek help informally and that increasing utilization will require addressing pragmatic barriers of time and cost. Assessing physician preferences for wellness-related initiatives may contribute to understanding the low utilization of formal mental health services among physicians, which can be determined using a BWS framework.

## Introduction

Physician burnout affects physicians worldwide and has negative implications for physician well-being and patient care, with a reported worldwide prevalence of 67% [1,2]. Multiple interventions have been proposed to address physician burnout; however, existing evidence is insufficient to recommend firm practical recommendations [3]. Some of this difficulty arises from the significant variation in reported burnout prevalence, which ranges from 0% to 80.5% [2]. This variation is poorly understood; there has been evidence that this can be attributed to inconsistency in measuring burnout and that burnout is experienced in a heterogeneous fashion among practice settings and specialties [4]. Calls have been proposed to better understand subjective and workplace factors that shape a physician’s experience outside the burnout construct [2,5,6].

Previous studies have attempted to identify factors associated with work-related stress and burnout. However, they have used traditional survey-based methodologies, such as Likert scales, focus groups, and ranking checklists [7,8]. Most surveys rely on these methods because of their ease of use and study design. However, these methods present multiple biases that affect statistical analysis and overall validity. Scale-use bias, the tendency for respondents to use rating scales in different ways, such as preferring to use higher or lower parts of the scale for all questions, can frequently be observed in studies using these scales [9]. There is also considerable debate as to whether Likert data are considered ordinal or interval, leading to the risk of flawed data interpretation [10]. Ranking checklists allow for improved discrimination among items but result in ordinal measures (which do not allow for mean calculations and relative differences between items) and become cognitively difficult when there are more than 7 items to rank [9].

It has been observed that physicians are low users of wellness resources to address burnout and other mental health issues such as depression and suicide [11]. Previous studies examining barriers to treatment have indicated that the most frequently cited issues are related to lack of time, lack of confidentiality, stigma, and preference to manage problems on their own [11,12]. Unfortunately, no published studies have examined whether physicians have differential attitudes toward mental health treatment options, such as individual therapy, group therapy, or peer support.

At our institution, we also encountered low use of wellness resources among physician residents in our internal medicine department with a desire to examine the barriers that contributed to this as well as their overall interest in these resources. We sought to use a novel technique to obtain this information.

Best-worst scaling (BWS) is a type of discrete choice experiment (DCE) based on economic choice modeling theory [13]. In this conceptual framework, individual and aggregate preferences to surveyed items can be developed by forcing respondents to choose from among two or more alternatives. BWS has been developed to resolve many of the biases associated with rating scales and ranking studies [9]. There is evidence that it allows for better item discrimination compared with rating scales in head-to-head comparisons, and BWS has recently been used to drive patient-centric health care decision-making by assessing patient preferences [13-16]. To date, no burnout-related studies have used this framework to investigate help-seeking preferences and barriers to using support services.

The primary goals of this study are to use the BWS framework to identify how residents prefer to seek help to prevent burnout and work-related stress and establish the relative importance of barriers to using commonly offered wellness interventions at our institution. We hoped to use this novel framework to better understand residents’ preferences to help-seeking and barriers to seeking help. None of the prior studies examining barriers to physician mental health utilization have used an economic preference framework to establish the relative importance of these items, which would be helpful in determining the most important barriers to address and which mental health services should be preferentially deployed from a policy-making standpoint. This information would be helpful to increase the overall utilization of mental health services and better address physician burnout.

## Methods

### What Is BWS?

BWS is a type of DCE based in economic theory. DCEs elicit respondent preferences for goods or services based on their stated intentions in hypothetical situations [17]. The term utility refers to the mathematical representation of preference, which, under microeconomic theory, assumes that decision makers will make choices that maximize the value of their utility function, subject to constrained resources [18].

**Figure 1:**
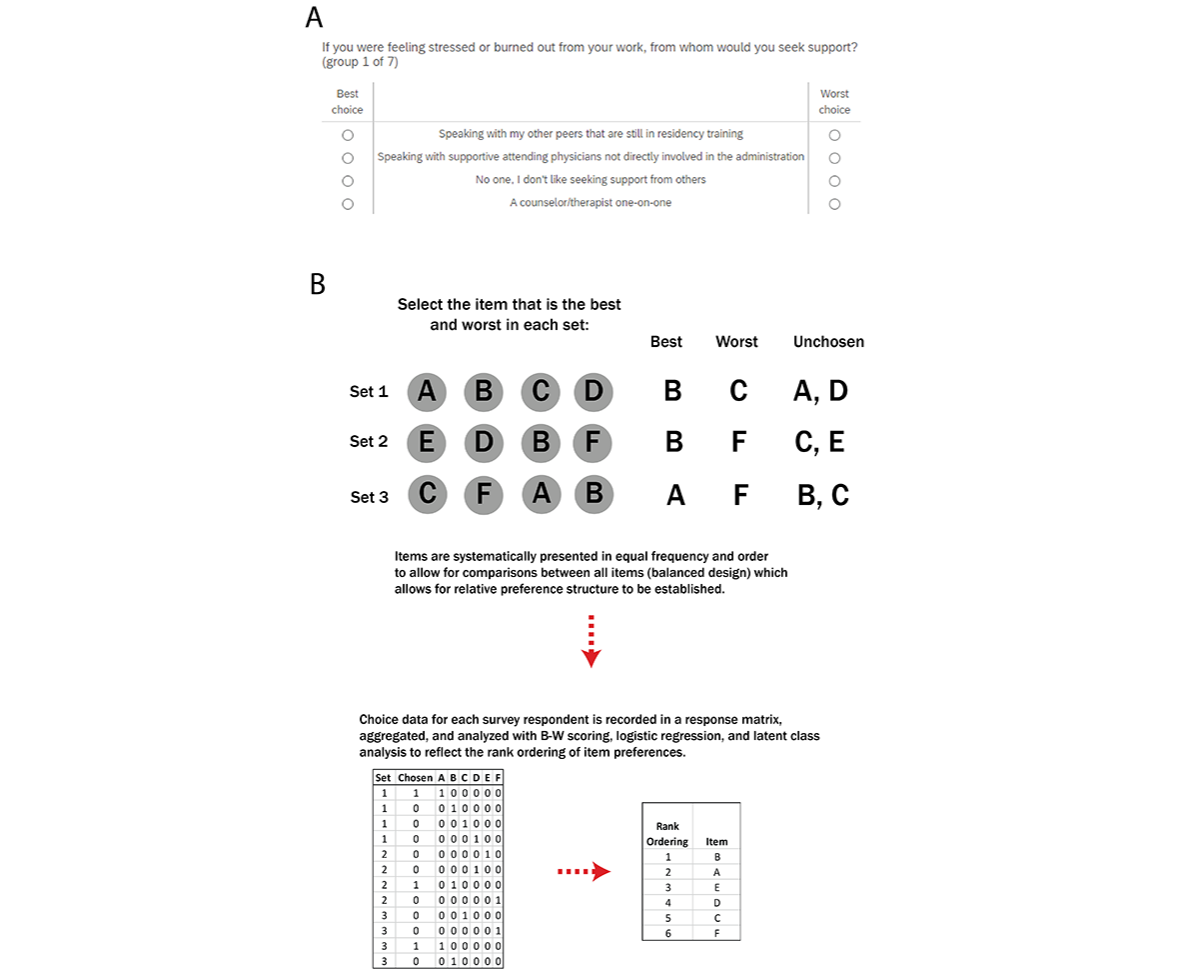
An example best-worst scaling object case question. Respondents choose one factor as the best choice and one factor as the worst choice in each question, with each factor systematically shown with all other factors in subsequent questions to allow for relative comparison.

There are 3 types of BWS surveys that differ in survey design complexity. More complex BWS survey types, such as profile or multiprofile case, allow for the comparison of factors with multiple attributes (eg, a medication with different prices, side effects, and modes of administration). The simplest version, the object case (also known as MaxDiff), was used for this study, which determines the relative value of a list of mutually exclusive objects [16]. An example of a BWS object case question is shown in Figure 1, where respondents are asked to identify the most preferred and least preferred item from a set of scenarios.

### Study Design

The first step in designing this BWS experiment was to determine the relevant factors to be included in the study. We initially used a comprehensive literature search to identify commonly cited barriers to wellness support and various help-seeking interventions [11,19,20]. We then worked with contributing authors ET and TMS, who serve as assistant program directors in the internal medicine residency program and psychiatry residency program, respectively, to elicit their experiences while working with resident physicians with regard to help-seeking interventions and frequently cited barriers to seeking care. AW, VR, TMS, and ET then worked together to optimize this list of factors such that the items were clearly defined, relevant, and did not have a singular factor that would not be universally selected as best or worst (item dominance), which are required for BWS study generation [17]. Item optimization was further refined in the pretesting stage, as described in the following section.

### Experimental Design

Inherent in BWS experimental design is the requirement to have systematic combinations of all surveyed items. This necessitates the need for a balanced design in frequency and orthogonality, where each surveyed factor needs to appear an equal number of times and equally with other factors, ensuring that each factor can be compared with all possible arrangements of other factors in the set. This was achieved using a balanced incomplete block design, a commonly used technique to design BWS sets and generated in JMP Pro 14 (SAS Institute Inc) [9,21]. Three BWS object case sets were created to assess preferences for each of the following factors: seeking support for work-related stress, barriers to using counseling, and barriers to using peer support groups. The three sets each tested seven factors, with every question in the set containing a subset of four factors, with a total of seven questions per set, which adhered to a balanced incomplete block design.

### Pretesting

After the initial study design, AW pretested the survey with a subset of 9 volunteer psychiatry residents. Adhering to a previous framework on BWS instrument development, we focused pretesting on the comprehension of the surveyed factors, whether there were omitted factors, the clarity of the BWS format, and the study purpose [22].

### Participants

Before study deployment, we worked with the Committee on Clinical Investigation for Beth Israel Deaconess Medical Center (BIDMC), the institutional review board for our institution, to receive an institutional review board exemption for this study. BIDMC is a teaching hospital in Boston, Massachusetts, with academic affiliations to Harvard Medical School. The internal medicine residency program is one of 13 Accreditation Council for Graduate Medical Education–accredited programs at the institution and comprises a total of 163 residents. It is a 3-year residency program that comprises resident physicians in their first postgraduate year of training (PGY-1) to their third postgraduate year of training (PGY-3). To be eligible for the survey, participants needed to be a resident physician between PGY-1 and PGY-3 years of training in the internal medicine program at BIDMC. Chief residents (who are in PGY-4 or PGY-5 of their training) were not invited, and the study did not include residents of other specialties.

In October 2020, residents in the BIDMC Internal Medicine residency were invited via email to participate in the study. This email contained a prospective agreement with consent and a link to the study, administered using Qualtrics Survey Software (SAP SE), emphasizing that participation in the study was voluntary and that data collected would not be individually identifiable. The demographic data collected included their age, postgraduate training year, and gender. These data were stored on Qualtrics Survey Software servers, which could only be accessed on an account that required two-factor encryption, for which only AW had access.

A total of 3 reminder emails were sent 1, 2, and 4 weeks after the study opened. After 6 weeks, the survey collection period ended. A US $5 Amazon gift card was given as compensation conditional to the completion of the survey.

### Statistical Analysis

A typical survey response rate for a web-based survey is 40%, which, using a margin of error calculation with our sample size, would have resulted in a margin of error of 9%. Given the desire to explore the novel nature of this study method and the possibility that it could result in highly discriminative utility values, we aimed for a 50% response rate, which would result in a margin of error of 8%. A BWS-specific power analysis was also conducted, which indicated that a 50% response rate (n=82) would have an 80% chance of detecting utility differences >0.118 at 95% CI; with a 40% response rate (n=65), we would be able to determine utility differences of 0.132 at 95% CI [23].

### Data Analysis

BWS data were analyzed using three techniques (Best-worst [B-W] scoring, multinomial logit [MNL], and latent class analysis [LCA]). For more details on multinomial logit and latent class analyses in this study, please refer to the Multimedia Appendices 1 and 2.

B-W scoring was conducted as described in previous BWS studies [13].

B-W scoring = ([number of times item selected best choice] – [number of times item selected worst]) / (*number of times surveyed factor displayed in the total survey*)

B-W scores have a range between −1.0 and +1.0, with scores closer to +1.0 having higher selected preference than lower-scoring options.

MNL and LCA modeling were performed using Lighthouse Studio 9.8 (Sawtooth Software). One-way analysis of variance with Tukey honest significant difference (HSD) posthoc test was performed using JMP Pro 14 to analyze the mean differences between factor preferences for survey respondents.

Rank ordering of factors was determined by sorting the raw utility values of the surveyed factors.

## Results

Of the 163 residents, the survey resulted in 77 (47.2% response rate) completing the study. This corresponds to an error margin of 8%, using a 95% CI. The age of the respondents ranged between 25 and 35 years, with 63% (49/77) of the total respondents being female. PGY-1s comprised 45% (35/77) of the total respondents, PGY-2s comprised 29% (22/77) of respondents, and PGY-3s comprised 26% (20/77) of respondents. These demographic characteristics are presented in Table 1.

When examining rank-ordered preferences for seeking support, the first-ranked factor was informally speaking with other resident peers, and the lowest ranking factor (#7) was not seeking help at all. Other high-ranking factors included informally speaking with friends and family for support (#2) and speaking with a counselor or therapist (#3). A total of 4 statistically significant groupings were found for the seven surveyed factors when comparing MNL utility scores with Tukey HSD posthoc testing. LCA optimally identified four classes with distinct preferences: *open to formal help* (39.4%), *not open to therapy* (26.8%), *open to isolating* (25.9%), and *formal help-seeking* (7.9%). The results are presented in Table 2.

Rank-ordered preferences for stated barriers to seeking counseling are shown in Table 3. The first-ranked factor was time, with the second-ranked factor being unwillingness to pay for counseling. The lowest ranking factors included feeling weak for seeing a therapist (#7), shame and embarrassment (#6), and not finding therapy helpful (#5). A total of 4 statistically significant groupings were found for the seven surveyed factors when comparing MNL utility scores with Tukey HSD posthoc testing. LCA optimally identified five classes with distinct preferences: *time/money* (49%), *time/money/don’t find it helpful* (20%), *confidentiality and future job concerns* (13%), *time/don’t find it helpful* (11%), and *high self-/social-stigma* (8%).

**Table 1:** Baseline characteristics (N=77).

Characteristics			Participants
**Age (years)**			
	Mean (SD)	28 (2)	
	Range	25-35	
**Gender, n (%)**			
	Female	49 (63)	
	Male	28 (37)	
**Training year, n (%)**			
	PGY^a^-1	35 (45)	
	PGY-2	22 (29)	
	PGY-3	20 (26)	

^a^PGY: postgraduate year of training.

**Table 2:** Rank-ordered resident preferences for seeking support for work-related stress and/or burnout (if you were feeling stressed or burned out from your work, from whom would you seek support?): frequency counts, best-worst scoring, multinomial logistic analysis, and latent class segmentation rank ordering.

Surveyed factors	Aggregate data						Latent class segmentation (rank order)			
	Number of times selected (maximum possible: 308)		Best-worst score	MNL^a^ utility score (SE)^b^	Rank order	Segment 1: “Open to formal help,” (39.4%^c^)		Segment 2: “Not open to therapy,” (26.8%)	Segment 3: “Open to isolating” (25.9%)	Segment 4: “Formal help-seeking“ (7.9%)
	Best choice	Worst choice								
Speaking with my other peers that are still in residency training	219	2	0.705	0.077 (0.15)^A^	1	2		1	2	1
Speaking with my family and friends outside of work	215	5	0.682	0.000 (N/A^d^)^A^	2	1		2	1	3
A counselor or therapist one-on-one	36	53	−0.055	−3.01 (0.211)^B^	3	3		6	4	2
Residency-sponsored peer support group, (like Intern Forum, but not necessarily just for interns)	30	49	−0.062	−3.088 (0.212)^B^	4	4		5	5	6
Speaking with supportive attending physicians not directly involved in the administration	5	71	−0.215	−3.404 (0.213)^B,C^	5	5		4	6	5
Speaking with my administration (chief residents or program directors)	23	130	−0.347	−3.919 (0.215)^C^	6	6		3	7	4
No one, I don’t like seeking support from others	11	229	−0.708	−5.043 (0.224)^D^	7	7		7	3	7

^a^MNL: multinomial logit.

^b^Four statistically significant groupings (A-D) were found for the 7 surveyed factors when comparing multinomial logit utility scores with Tukey honest significant difference posthoc testing. Mean utility scores followed by the same letter did not differ significantly (Tukey honest significant difference test, *P*>.05); exact *P* values for multiple comparisons are shown in Table S1 in Multimedia Appendix 1.

^c^The latent number of groups displayed was based on the lowest Bayesian information criterion. Owing to the probabilistic nature of the latent class method, respondents do not wholly belong to one group or another, although most respondents (74/77, 96%) had >90% probability of membership to a single group.

^d^N/A: not applicable.

**Table 3:** Rank-ordered resident preferences for stated barriers to seeking counseling (if the residency program offered one-on-one counseling for stress and burnout from work, what do you think could affect your participation?).

Surveyed factors	Aggregate data										Latent class segmentation (rank order)									
	Number of times selected (maximum possible: 308)			Best-worst score		MNL^a^ utility score (SD)^b^		Rank order		Segment 1: “Time/ money,” (49%)			Segment 2 “Time/ money/ don’t find it helpful,” (20%)		Segment 3: “confidentiality and future job concerns” (13%)		Segment 4: “Time/ don’t find it helpful” (11%)		Segment 5: “High self-/ social-stigma” (8%)	
	Most important	Least important																		
I wouldn’t have enough time	231	14	0.705		2.528 (0.150)^A^		1		1			1		4		1		1		
I wouldn’t want to pay for it	109	64	0.146		1.009 (0.133)^B^		2		2			2		3		7		7		
I'm concerned that seeing a therapist will reflect poorly on my standing as a resident or impact my future job opportunities	58	75	−0.056		0.454 (0.131)^C^		3		4			7		1		4		4		
I’m concerned about the confidentiality of talking about my issues to a therapist	53	78	−0.081		0.416 (0.133)^C^		4		3			6		2		3		5		
I don’t think it would help for addressing my wellness	56	81	−0.081		0.406 (0.132)^C^		5		5			3		6		2		6		
I would be ashamed or embarrassed if my peers knew I was seeing a therapist	16	89	−0.237		0 (N/A^c^)^C,D^		6		6			5		5		6		2		
I would think I’m a weak person for seeing a therapist for stress or burnout	16	138	−0.396		−0.407 (0.129)^D^		7		7			4		7		5		3		

^a^MNL: multinomial logit.

^b^Four statistically significant groupings (A-D) were found for the 7 surveyed factors when comparing multinomial logit utility scores with Tukey honest significant difference posthoc testing. Mean utility scores followed by the same letter did not differ significantly (Tukey honest significant difference test, *P*>.05); exact *P* values for multiple comparisons are shown in Table S1 in Multimedia Appendix 1.

^c^N/A: not applicable.

Stated barriers for participation in the resident peer-support group are shown in Table 4. The first ranking factor was not having enough time during the workday. Other top-ranking factors included being too fatigued (#2) and being off-site/on vacation (#3). The lowest ranking factors included not liking classmates (#7), embarrassing oneself in front of one’s peers (#6), and fearing that what one shared would reflect poorly of oneself as a physician (#5). A total of 5 statistically significant groupings were found for the seven surveyed factors when comparing MNL utility scores with Tukey HSD posthoc testing. LCA optimally identified four classes with distinct preferences: *time/too tired* (32%), *time/too tired/don’t find it helpful* (26%), time/too tired/don’t want to share (22%), and *time/off-site* (20%).

**Table 4:** Rank-ordered resident preferences for stated barriers for participation in a resident peer support group. (Thinking back to intern year, select the most and least significant factors that affected your participation in the residency-provided peer support group [Intern Forum] that occurred during the day for work-related stress and/or burnout).

Surveyed factors	Aggregate data						Latent class segmentation (rank order)			
	Number of times selected (maximum possible: 308)		Best-worst score	MNL^a^ utility score (SE)^b^	Rank order	Segment 1: “Time/too tired,“ (32%)		Segment 2: “Time/too tired/don’t find it helpful,” (26%)	Segment 3: “Time/too tired/Don’t want to share,“ (22%)	Segment 4: “Time/off-site,” (20%)
	Most important	Least important								
I don't have enough time during the workday	187	8	0.581	2.125 (0.150)^A^	1	1		2	1	2
I’m too tired	113	20	0.302	1.386 (0.147)^B^	2	2		3	2	3
I'm off-site, post-call, or on vacation	86	48	0.12	0.780 (0.139)^C^	3	3		4	6	1
I don't think it will help with addressing my wellness	79	42	0.12	0.858 (0.145)^B,C^	4	4		1	5	6
I'm concerned that what I share will reflect poorly of me as a resident and physician	36	79	−0.139	0.000 (N/A^c^)^D^	5	6		5	4	4
I don't want to embarrass myself in front of my peers	36	85	−0.159	−0.017 (0.142)^D^	6	5		6	3	5
I don't like my classmates	2	257	−0.828	−2.154 (0.170)^E^	7	7		7	7	7

^a^MNL: multinomial logit.

^b^Five statistically significant groupings (A-E) were found for the 7 surveyed factors when comparing multinomial logit utility scores with Tukey honest significant difference posthoc testing. Mean utility scores followed by the same letter did not differ significantly (Tukey honest significant difference test, *P*>.05); exact *P* values for multiple comparisons are shown in Table S1 in Multimedia Appendix 1.

^c^N/A: not applicable.

Each factor was shown a total of four times for each of the 77 respondents, resulting in a maximum possibility of 308 best or worst choice selections. For every question, respondents had the option of selecting two of the four factors as either the best or worst choice, leaving the remaining two factors unselected.

B-W scores have a range between −1.0 and +1.0, with scores closer to +1.0 having higher selected preference than lower-scoring options.

The utility score of one item is required to be set to zero because of linear dependency constraints (speaking with my family and friends outside of work). The mean utility scores followed by the same letter did not differ significantly (Tukey HSD test; *P*>.05); exact *P* values for multiple comparisons are shown in Table S1 in Multimedia Appendix 1.

The latent number of groups displayed was based on the lowest Bayesian information criterion. Owing to the probabilistic nature of the latent class method, respondents do not wholly belong to one group or another, although most respondents (74/77, 96%) had >90% probability of membership to a single group. Segment percentages refer to respondents that have been assigned to their respective segments using latent class segmentation over the total respondent population (n=77).

Each factor was shown a total of four times to each of the 77 respondents, resulting in a maximum possibility of 308 best or worst choice selections.

The mean utility scores followed by the same letter did not differ significantly (Tukey HSD test; *P*>.05); exact *P* values for multiple comparisons are shown in Table S2 in Multimedia Appendix 1.

Latent class MNL with 5 groups is shown. Owing to the probabilistic nature of the latent class method, respondents do not wholly belong to one group or another, although most respondents (73/77, 95%) had >90% probability of membership to a single group. Segment percentages refer to respondents that have been assigned to their respective segments using latent class segmentation over the total respondent population (n=77)

Each factor was shown a total of four times to each of the 77 respondents, resulting in a maximum possibility of 308 best or worst choice selections.

The mean utility scores followed by the same letter did not differ significantly (Tukey HSD test; *P*>.05); exact *P* values for multiple comparisons are shown in Table S3 in Multimedia Appendix 1.

Latent class MNL with 4 groups is shown. Owing to the probabilistic nature of the latent class method, respondents do not wholly belong to one group or another, although most respondents (68/77, 88%) had >90% probability of membership to a group. Segment percentages refer to respondents that have been assigned to their respective segments using latent class segmentation over the total respondent population (n=77).

## Discussion

### Principal Findings

To our knowledge, this is the first known survey on resident burnout and wellness support that uses a BWS methodology. This framework allows resident program leadership to gather specific information on what residents value when it comes to seeking help and relevant barriers. Overall, surveyed residents most preferred to seek emotional support informally from their resident peers and with friends and family when presented with other options of formal mental health support or administrative support. When examining self-stated barriers to using therapy, residents most frequently cited pragmatic barriers of time and money rather than those related to mental health stigma.

Our study findings also reflect prior studies where time was the most frequently cited barrier to using mental health services among resident physicians [11,24]. Future efforts designed to increase use should thus consider how to best mitigate this barrier, such as dedicating specific time in a resident's schedule to allow for medical appointments or to assess the viability of using telehealth services for these purposes, which would allow for more flexibility on a resident's schedule [20,25]. Although the preference for informal support over formal counseling among physicians would need to be reestablished in the future with a larger sample size, a recent BWS study examining help-seeking preferences for mental health concerns among college students also similarly reflected a preference toward informal sources of support through friends and family over formal counseling [26]. As such, when considering the low overall utilization rate of mental health services among physicians, it may also be worth considering that preferences for informal support over formal counseling exist among the general population and that this may not represent a particularly unique issue among physicians, although this is certainly worth investigating in the future.

The BWS framework also allows for the use of latent class modeling, identifying segments of residents that share similar preferences. Although these findings are difficult to generalize based on our small study size, the identification of segments of residents that mostly prefer formal counseling, prefer to deal with problems on their own, or prefer informal supports represent potential phenotypes of residents that warrant future investigation, such as whether the frequency of these groupings is stable across differing institutions, specialties, and countries. Although future studies showing similar frequencies and phenotypes would aid in the generalizability of our study findings regarding physician resident attitudes on barriers to using mental health and seeking help, it cannot be expected that preferences will be identical, given the aforementioned variations in clinical settings and the variety of personality traits and personal values that physicians possess [27]. What could also be more useful for policy makers would be using repeated assessments at singular institutions of BWS-based studies across time to assess resident physician preferences regarding wellness initiatives longitudinally, which would also aid in determining the impact of potential interventions designed to encourage wellness initiative utilization.

### Limitations

The external validity of this study was limited by the singular specialty surveyed. Respondents only included 47% of the total internal medicine residency and may not reflect the aggregate preferences of all medical residents. There is a possibility that nonresponders could have had differing preferences from those that responded; given how a previous study examining physician response rates to web-based surveys cited lack of time as a primary reason for not responding to surveys, there is a possibility that the magnitude of time as a barrier to seeking help was understated [28]. As we did not survey other residents in other specialties, we cannot determine whether these preferences are generalizable to all residents or if they differ by specialty. However, we believe that the surveyed factors related to help-seeking barriers are universal to many residents; notably, most residents struggle with being able to take time to seek formal counseling, and issues of self-stigma and social stigma, when it comes to receiving mental health services, are not solely limited to our residency program. Furthermore, mental health interventions such as individual therapy and peer support are commonly offered wellness supports in many institutions. There is no reason to believe that these barriers are not encountered by other resident physicians; however, it would be interesting to repeat this survey in other specialties to observe the generalizability of our collected preferences. Although our study sample contained a higher proportion of female respondents, raising a concern that data would be skewed, we did not find meaningful differences in preference choices by gender, as shown in Table S5 in Multimedia Appendix 1.

### Conclusions

To our knowledge, this is the first known study that uses a BWS approach to establish resident preferences for seeking support. [2]. In our study, understanding physician preferences for seeking help and stated barriers to seeking formal help was useful in generating potential reasons why these resources have been underutilized at our institution, in particular, a preference for informal help and strongly cited barriers related to time constraints.

By using a BWS framework for wellness initiative implementation, institutions can accurately assess the relevant barriers and demand for wellness services among their clinician population, for which these preferences will likely vary because of variations in practice settings, clinician specialties, and institutional culture. The design of BWS studies allows for repeated measurements of surveyed factors in a more efficient study design, allowing for more reliable measures of preferences of these factors on an individual respondent level in a survey that takes only a few minutes to complete. In contrast, traditional surveys using Likert scales cannot establish these preferences as they do not collect repeated measures of these data, do not force respondents to choose between surveyed factors, and are subject to scale-use bias, allowing policy makers to better understand how respondents value the surveyed factors.

This method may save money and time by reducing the likelihood of institutions implementing wellness initiatives that turn out to be underutilized because of unforeseen barriers and unique preferences for services. Physician residents are not a homogenous population where a singular intervention will be universally helpful to improve their wellness, as suggested by the lack of evidence of such a universal intervention and wide range of burnout prevalence among practice settings.

Although the design of BWS studies requires some understanding of the underlying discrete choice theory, several commonly used statistical platforms allow for the design and analysis of these studies (JMP, SAS, Qualtrics Survey Software, STATA, and R). Thus, the BWS framework provides an accessible opportunity to create personalized approaches to addressing clinician wellness.

## Appendix

Multimedia Appendix 1 Additional data and information provided on statistical interpretation of best-worst scaling data.

Multimedia Appendix 2 Full respondent survey.

## Abbreviations

BIDMC: Beth Israel Deaconess Medical Center
B-W: best-worst
BWS: best-worst scaling
DCE: discrete choice experiment
HSD: honest significant difference
LCA: latent class analysis
MNL: multinomial logit
PGY: postgraduate year of training
