# Supplementary material for: Self-Reported Preferences for Help-Seeking and Barriers to Using Mental Health Supports Among Internal Medicine Residents: Exploratory Use of an Econometric Best-Worst Scaling Framework for Gathering Physician Wellness Preferences
Source: JMIR Med Educ. 2021 Oct 6;7(4):e28623. doi: 10.2196/28623 (PMC8529465; doi:10.2196/28623)
Supplement: Multimedia Appendix 1 [file mededu_v7i4e28623_app1.docx]

# Supplemental Methods: Multinomial Logit and Latent Class Analyses

While B-W scoring may be the simplest way of reporting aggregate data, BWS survey responses should best be interpreted as choices with respondents following random utility theory (utility can be derived from the relative frequency that an individual prefers an item over another with an additional random error component); this can be best modeled using multinomial logistic modeling of observed pairs of best- and worst- choices. Choices are coded with a dummy variable, where best choices are codified as scoring “1”, worst choices as “-1”, and unselected choices as “0.”

Logistic regression is conducted with the choice dummy variable as the outcome variable and the surveyed items as predictor variables. By running a logistic regression using aggregate choice data, coefficients for each item are calculated which represent each item’s utility score. This can not only be used for rank ordering, but can be transformed into ratio scoring to represent the probability of an item being chosen over the other items. One item’s utility score is required to be set to zero in the regression analysis due to linear dependency constraints.

Utility scores can be transformed into probability ratio scores using the logit choice rule, which specifies the probability of choosing the *i*^th^ item as best from a set containing *i* through *j* times is equal to: $\text{P}_{\text{i}}=e^{U_{i}}/\sum e^{U_{ij}}$, with $e^{u_{i}}=\text{the antilog of the utility for item }\text{i}$.

Latent class modeling is a mixture modeling technique used to identify unobserved heterogeneity in a population [28]. This can be helpful in choice modeling to identify sub-populations of individuals that have distinct preference profiles [29]. These modeling methods rely on probabilistic modeling for which fit statistics such as Akaike information criterion (AIC) and Bayesian information criterion (BIC) can be used to evaluate how well each model fits the data set [30].

Latent class analysis was conducted in Sawtooth Software with default options selected as follows: Minimum group:2, Maximum group: 5; number of replications for each solution: 5; maximum number of iterations: 500; convergence limit for log-likelihood: 0.01. Several measures of fit are provided with each latent class analysis: log likelihood, percent certainty, consistent Akaike Information Criterion (CAIC), Chi Square, Relative Chi Square, BIC, and AIC. For full explanation of these fit statistics, please refer to the latent class technical paper by Sawtooth Software [31]. Though there is no universal statistical criterion to determine the optimal number of segments, the most widely used metric to determine best fit is by minimizing BIC values [28]. Full BIC values from LCA are shown in Supplemental Table 4 in Multimedia Appendix 1.

Supplemental Table 1. Significance values from multiple comparisons of factor utilities using Tukey’s HSD test for BWS Set 1: Resident Preferences for Seeking Support for Work-Related Stress and/or Burnout

| **Level** | **- Level** | **p-Value** |
| --- | --- | --- |
| I don't have enough time during the workday | I don't like my classmates | <.0001* |
| I'm too tired | I don't like my classmates | <.0001* |
| I don't think it will help with addressing my wellness | I don't like my classmates | <.0001* |
| I'm off-site, post-call, or on vacation | I don't like my classmates | <.0001* |
| I'm concerned that what I share will reflect poorly of me as a resident and physician | I don't like my classmates | <.0001* |
| I don't have enough time during the workday | I don't want to embarrass myself in front of my peers | <.0001* |
| I don't want to embarrass myself in front of my peers | I don't like my classmates | <.0001* |
| I don't have enough time during the workday | I'm concerned that what I share will reflect poorly of me as a resident and physician | <.0001* |
| I'm too tired | I don't want to embarrass myself in front of my peers | <.0001* |
| I'm too tired | I'm concerned that what I share will reflect poorly of me as a resident and physician | <.0001* |
| I don't have enough time during the workday | I'm off-site, post-call, or on vacation | <.0001* |
| I don't have enough time during the workday | I don't think it will help with addressing my wellness | <.0001* |
| I don't think it will help with addressing my wellness | I don't want to embarrass myself in front of my peers | 0.0002* |
| I don't think it will help with addressing my wellness | I'm concerned that what I share will reflect poorly of me as a resident and physician | 0.0003* |
| I'm off-site, post-call, or on vacation | I don't want to embarrass myself in front of my peers | 0.0010* |
| I'm off-site, post-call, or on vacation | I'm concerned that what I share will reflect poorly of me as a resident and physician | 0.0015* |
| I don't have enough time during the workday | I'm too tired | 0.0033* |
| I'm too tired | I'm off-site, post-call, or on vacation | 0.0331* |
| I'm too tired | I don't think it will help with addressing my wellness | 0.0999 |
| I don't think it will help with addressing my wellness | I'm off-site, post-call, or on vacation | 0.9997 |
| I'm concerned that what I share will reflect poorly of me as a resident and physician | I don't want to embarrass myself in front of my peers | 1.0000 |

Supplemental Table 2. Significance values from multiple comparisons of factor utilities using Tukey’s HSD test for BWS Set 2: Stated Barriers to Seeking Counseling

| **Level** | **- Level** | **p-Value** |
| --- | --- | --- |
| I wouldn't have enough time | I would think I’m a weak person for seeing a therapist for stress or burnout | <.0001* |
| I wouldn't have enough time | I don’t think it would help for addressing my wellness | <.0001* |
| I wouldn't have enough time | I’m concerned about the confidentiality of talking about my issues to a therapist | <.0001* |
| I wouldn't have enough time | I'm concerned that seeing a therapist will reflect poorly on my standing as a resident or impact my future job opportunities | <.0001* |
| I wouldn’t want to pay for it | I would think I’m a weak person for seeing a therapist for stress or burnout | <.0001* |
| I wouldn't have enough time | I wouldn’t want to pay for it | <.0001* |
| I'm concerned that seeing a therapist will reflect poorly on my standing as a resident or impact my future job opportunities | I would think I’m a weak person for seeing a therapist for stress or burnout | 0.0031* |
| I’m concerned about the confidentiality of talking about my issues to a therapist | I would think I’m a weak person for seeing a therapist for stress or burnout | 0.0054* |
| I wouldn’t want to pay for it | I don’t think it would help for addressing my wellness | 0.0074* |
| I don’t think it would help for addressing my wellness | I would think I’m a weak person for seeing a therapist for stress or burnout | 0.1579 |
| I wouldn’t want to pay for it | I’m concerned about the confidentiality of talking about my issues to a therapist | 0.1918 |
| I wouldn’t want to pay for it | I'm concerned that seeing a therapist will reflect poorly on my standing as a resident or impact my future job opportunities | 0.2604 |
| I'm concerned that seeing a therapist will reflect poorly on my standing as a resident or impact my future job opportunities | I don’t think it would help for addressing my wellness | 0.7666 |
| I’m concerned about the confidentiality of talking about my issues to a therapist | I don’t think it would help for addressing my wellness | 0.8473 |
| I'm concerned that seeing a therapist will reflect poorly on my standing as a resident or impact my future job opportunities | I’m concerned about the confidentiality of talking about my issues to a therapist | 1.0000 |

Supplemental Table 3. Significance values from multiple comparisons of factor utilities using Tukey’s HSD test for BWS Set 3: Stated Barriers to Participation in Peer Support Group

| **Level** | **- Level** | **p-Value** |
| --- | --- | --- |
| I don't have enough time during the workday | I don't like my classmates | <.0001* |
| I'm too tired | I don't like my classmates | <.0001* |
| I don't think it will help with addressing my wellness | I don't like my classmates | <.0001* |
| I'm off-site, post-call, or on vacation | I don't like my classmates | <.0001* |
| I'm concerned that what I share will reflect poorly of me as a resident and physician | I don't like my classmates | <.0001* |
| I don't have enough time during the workday | I don't want to embarrass myself in front of my peers | <.0001* |
| I don't want to embarrass myself in front of my peers | I don't like my classmates | <.0001* |
| I don't have enough time during the workday | I'm concerned that what I share will reflect poorly of me as a resident and physician | <.0001* |
| I'm too tired | I don't want to embarrass myself in front of my peers | <.0001* |
| I'm too tired | I'm concerned that what I share will reflect poorly of me as a resident and physician | <.0001* |
| I don't have enough time during the workday | I'm off-site, post-call, or on vacation | <.0001* |
| I don't have enough time during the workday | I don't think it will help with addressing my wellness | <.0001* |
| I don't think it will help with addressing my wellness | I don't want to embarrass myself in front of my peers | 0.0002* |
| I don't think it will help with addressing my wellness | I'm concerned that what I share will reflect poorly of me as a resident and physician | 0.0003* |
| I'm off-site, post-call, or on vacation | I don't want to embarrass myself in front of my peers | 0.0010* |
| I'm off-site, post-call, or on vacation | I'm concerned that what I share will reflect poorly of me as a resident and physician | 0.0015* |
| I don't have enough time during the workday | I'm too tired | 0.0033* |
| I'm too tired | I'm off-site, post-call, or on vacation | 0.0331* |
| I'm too tired | I don't think it will help with addressing my wellness | 0.0999 |
| I don't think it will help with addressing my wellness | I'm off-site, post-call, or on vacation | 0.9997 |
| I'm concerned that what I share will reflect poorly of me as a resident and physician | I don't want to embarrass myself in front of my peers | 1.0000 |

Supplemental Table 4. Latent Class Analysis Statistical Information Criterion for BWS Sets. Lowest BIC for each BWS Set was used to determine optimal number of groups per set.

| **BWS Set 1: Help-Seeking Preferences** | |
| --- | --- |
| Groups | **BIC** |
| 2 | 1513.51 |
| 3 | 1466.71 |
| 4 | **1448.63** |
| 5 | 1451.74 |
| **BWS Set 2: Barriers to Seeking Support** | |
| Groups | **BIC** |
| 2 | 2217.14 |
| 3 | 2156.94 |
| 4 | 2132.49 |
| 5 | **2101.06** |
| 6 | 2110.53 |
| **BWS Set 3: Barriers to Participation in Resident Peer Support Group** | |
| Groups | **BIC** |
| 2 | 1946.37 |
| 3 | 1882.58 |
| 4 | **1868.85** |
| 5 | 1870.18 |

Supplemental Table 5: Gender Differences in BWS Sets 1,2,3.

|  | Female (n=49) | Male (n=28) |  |
| --- | --- | --- | --- |
| Surveyed Factor | Zero-Centered Interval Score (std error) | Zero-Centered Interval Score (std error) | *P*-value |
| **BWS Set 1: Help-Seeking Preferences** | | |  |
| Speaking with my family and friends outside of work | 41.90 (2.37) | 36.91 (2.53) | *P<*.18 |
| Speaking with my other peers that are still in residency training | 41.51 (1.49) | 42.22 (3.05) | *P*<.82 |
| A counselor/therapist one-on-one | -5.48 (2.07) | -2.89 (3.63) | *P*<.51 |
| Residency-sponsored peer support group | -6.14 (1.51) | -7.16 (2.30) | *P*<.70 |
| Speaking with supportive attending physicians not directly involved in the administration | -11.70 (1.57) | -11.47 (2.60) | *P*<.94 |
| Speaking with my administration [chief residents/program directors] | -19.37 (2.27) | -18.89 (3.46) | *P*<.90 |
| No one, I don't like seeking support from others | -40.73 (2.98) | -38.73 (4.44) | *P*<.70 |
| **BWS Set 2: Barriers to Utilizing Counseling** | | |  |
| I wouldn't have enough time | 53.34 (3.12) | 41.88 (6.18) | *P*<.07 |
| I wouldn’t want to pay for it | 10.24 (5.19) | 10.13 (6.84) | *P*<.99 |
| I’m concerned about the confidentiality of talking about my issues to a therapist | -5.57 (3.37) | -4.96 (4.18) | *P*<.91 |
| I'm concerned that seeing a therapist will reflect poorly on my standing as a resident or impact my future job opportunities | -7.68 (3.26) | -1.69 (5.70) | *P*<.33 |
| I don’t think it would help for addressing my wellness | -8.33 (2.97) | -7.03 (4.96) | *P*<.81 |
| I would be ashamed or embarrassed if my peers knew I was seeing a therapist | -17.07 (1.70) | -15.51 (2.86) | *P*<.62 |
| I would think I’m a weak person for seeing a therapist for stress or burnout | -24.93 (2.27) | -22.83 (3.74) | *P*<.61 |
| **BWS Set 3: Barriers to Utilizing Peer Support Group** | | |  |
|  |  |  |  |
| I don't have enough time during the workday | 35.28 (2.32) | 34.66 (2.77) | *P<*.87 |
| I'm too tired | 20.86 (3.02) | 20.58 (2.73) | *P*<.95 |
| I'm off-site, post-call, or on vacation | 7.94 (3.38) | 5.80 (5.07) | *P*<.72 |
| I don't think it will help with addressing my wellness | 7.55 (4.01) | 10.09 (4.79) | *P*<.69 |
| I'm concerned that what I share will reflect poorly of me as a resident and physician | -9.98 (3.04) | -9.83 (2.53) | *P*<.97 |
| I don't want to embarrass myself in front of my peers | -10.49 (2.78) | -7.58 (3.71) | *P*<.53 |
| I don't like my classmates | -51.16 (1.85) | -53.73 (1.37) | *P*<.34 |
