# Supplementary material for: Self-Reported Preferences for Help-Seeking and Barriers to Using Mental Health Supports Among Internal Medicine Residents: Exploratory Use of an Econometric Best-Worst Scaling Framework for Gathering Physician Wellness Preferences
Source: JMIR Med Educ. 2021 Oct 6;7(4):e28623. doi: 10.2196/28623 (PMC8529465; doi:10.2196/28623)
Supplement: Multimedia Appendix 2 [file mededu_v7i4e28623_app2.pdf]

Medicine Resident Preferences for Seeking Help

Demographics

Upon completion of this survey you will receive a $5 Amazon gift card if you provide a working BIDMC email address. This email will be de-linked from your survey responses to preserve anonymity.

What is your training year?

- PGY1
- PGY2
- PGY3
- PGY4 / PGY5

What is your gender?

- Male
- Female
- Non-binary
- Prefer not to say

What is your age?

Seeking Support

For each group of factors listed, please select a best (left button) and worst choice (right button). (I.E. every question should have two buttons selected)

Note: The redundancy of the questions is intentional and necessary for the study design.

If you were feeling stressed or burned out from your work, from whom would you seek support? (group 1 of 7)

Best choice		Worst choice
O	Speaking with my other peers that are still in residency training	O
O	Speaking with supportive attending physicians not directly involved in the administration	O
O	No one, I don't like seeking support from others	O

Best choice		Worst choice
○	A counselor/therapist one-on-one	○

If you were feeling stressed or burned out from your work, from whom would you seek support? (group 2 of 7)

Best choice		Worst choice
○	A counselor/therapist one-on-one	○
○	Residency-sponsored peer support group (like Intern Forum, but not necessarily just for interns)	○
○	Speaking with my administration [chief residents/program directors]	○
○	No one, I don't like seeking support from others	○

If you were feeling stressed or burned out from your work, from whom would you seek support? (group 3 of 7)

Best choice		Worst choice
○	Speaking with my administration [chief residents/program directors]	○
○	No one, I don't like seeking support from others	○
○	Speaking with my family and friends outside of work	○
○	Speaking with supportive attending physicians not directly involved in the administration	○

If you were feeling stressed or burned out from your work, from whom would you seek support? (group 4 of 7)

Best choice		Worst choice
○	No one, I don't like seeking support from others	○
○	Speaking with my family and friends outside of work	○
○	Speaking with my other peers that are still in residency training	○

Best choice		Worst choice
○	Residency-sponsored peer support group, (like Intern Forum, but not necessarily just for interns)	○

If you were feeling stressed or burned out from your work, from whom would you seek support? (group 5 of 7)

Best choice		Worst choice
○	Residency-sponsored peer support group	○
○	A counselor/therapist one-on-one	○
○	Speaking with supportive attending physicians not directly involved in the administration	○
○	Speaking with my family and friends outside of work	○

If you were feeling stressed or burned out from your work, from whom would you seek support? (group 6 of 7)

Best choice		Worst choice
○	Speaking with my family and friends outside of work	○
○	Speaking with my administration [chief residents/program directors]	○
○	A counselor/therapist one-on-one	○
○	Speaking with my other peers that are still in residency training	○

If you were feeling stressed or burned out from your work, from whom would you seek support? (group 7 of 7)

Best choice		Worst choice
○	Speaking with supportive attending physicians not directly involved in the administration	○
○	Speaking with my other peers that are still in residency training	○
○	Residency-sponsored peer support group (like Intern Forum, but not necessarily just for interns)	○

Best choice		Worst choice
○	Speaking with my administration [chief residents/program directors]	○

Social Outings Preferences

For each group of factors listed, please select the most significant (left button) and least significant barrier (right button). (I.E. every question should have two buttons selected)

Note: The redundancy of the questions is intentional and necessary for the study design.

If you weren't seeing a therapist and the residency program offered access to one-on-one counseling for work-related stress, what do you think could affect your participation? (group 1 of 7)

Most significant barrier		Least significant barrier
○	I'm concerned that seeing a therapist will reflect poorly on my standing as a resident or impact my future job opportunities	○
○	I would think I’m a weak person for seeing a therapist for stress or burnout	○
○	I wouldn’t want to pay for it	○
○	I wouldn't have enough time	○

If you weren't seeing a therapist and the residency program offered access to one-on-one counseling for work-related stress, what do you think could affect your participation? (group 2 of 7)

Most significant barrier		Least significant barrier
○	I wouldn't have enough time	○
○	I’m concerned about the confidentiality of talking about my issues to a therapist	○
○	I don’t think it would help for addressing my wellness	○
○	I wouldn’t want to pay for it	○

If you weren't seeing a therapist and the residency program offered access to one-on-one counseling for work-related stress, what do you think could affect your participation? (group 3 of 7)

Most significant barrier		Least significant barrier

Most significant barrier		Least significant barrier
○	I don’t think it would help for addressing my wellness	○
○	I wouldn’t want to pay for it	○
○	I would be ashamed or embarrassed if my peers knew I was seeing a therapist	○
○	I would think I’m a weak person for seeing a therapist for stress or burnout	○

If you weren't seeing a therapist and the residency program offered access to one-on-one counseling for work-related stress, what do you think could affect your participation? (group 4 of 7)

Most significant barrier		Least significant barrier
○	I wouldn’t want to pay for it	○
○	I would be ashamed or embarrassed if my peers knew I was seeing a therapist	○
○	I'm concerned that seeing a therapist will reflect poorly on my standing as a resident or impact my future job opportunities	○
○	I’m concerned about the confidentiality of talking about my issues to a therapist	○

If you weren't seeing a therapist and the residency program offered access to one-on-one counseling for work-related stress, what do you think could affect your participation? (group 5 of 7)

Most significant barrier		Least significant barrier
○	I’m concerned about the confidentiality of talking about my issues to a therapist	○
○	I wouldn't have enough time	○
○	I would think I’m a weak person for seeing a therapist for stress or burnout	○

Most significant barrier		Least significant barrier
○	I would be ashamed or embarrassed if my peers knew I was seeing a therapist	○

If you weren't seeing a therapist and the residency program offered access to one-on-one counseling for work-related stress, what do you think could affect your participation? (group 6 of 7)

Most significant barrier		Least significant barrier
○	I would be ashamed or embarrassed if my peers knew I was seeing a therapist	○
○	I don’t think it would help for addressing my wellness	○
○	I wouldn't have enough time	○
○	I'm concerned that seeing a therapist will reflect poorly on my standing as a resident or impact my future job opportunities	○

If you weren't seeing a therapist and the residency program offered access to one-on-one counseling for work-related stress, what do you think could affect your participation? (group 7 of 7)

Most significant barrier		Least significant barrier
○	I would think I’m a weak person for seeing a therapist for stress or burnout	○
○	I'm concerned that seeing a therapist will reflect poorly on my standing as a resident or impact my future job opportunities	○
○	I’m concerned about the confidentiality of talking about my issues to a therapist	○
○	I don’t think it would help for addressing my wellness	○

Select the most and least significant factors that affected your participation in the residency-provided peer support group during intern year (Intern Forum) (group 1 of 7)

Most significant factor		Least significant factor

Most significant factor		Least significant factor
○	I don't think it will help with addressing my wellness	○
○	I don't like my classmates	○
○	I'm off-site, post-call, or on vacation	○
○	I don't have enough time during the workday	○

Select the most and least significant factors that affected your participation in the residency-provided peer support group during intern year (Intern Forum) (group 2 of 7)

Most significant factor		Least significant factor
○	I don't have enough time during the workday	○
○	I'm too tired	○
○	I don't want to embarrass myself in front of my peers	○
○	I'm off-site, post-call, or on vacation	○

Select the most and least significant factors that affected your participation in the residency-provided peer support group during intern year (Intern Forum) (group 3 of 7)

Most significant factor		Least significant factor
○	I don't want to embarrass myself in front of my peers	○
○	I'm off-site, post-call, or on vacation	○
○	I'm concerned that what I share will reflect poorly of me as a resident and physician	○
○	I don't like my classmates	○

Select the most and least significant factors that affected your participation in the residency-provided peer support group during intern year (Intern Forum) (group 4 of 7)

Most significant factor		Least significant factor
○	I'm off-site, post-call, or on vacation	○
○	I'm concerned that what I share will reflect poorly of me as a resident and physician	○
○	I don't think it will help with addressing my wellness	○

Most significant factor		Least significant factor
○	I'm too tired	○

Select the most and least significant factors that affected your participation in the residency-provided peer support group during intern year (Intern Forum) (group 5 of 7)

Most significant factor		Least significant factor
○	I'm too tired	○
○	I don't have enough time during the workday	○
○	I don't like my classmates	○
○	I'm concerned that what I share will reflect poorly of me as a resident and physician	○

Select the most and least significant factors that affected your participation in the residency-provided peer support group during intern year (Intern Forum) (group 6 of 7)

Most significant factor		Least significant factor
○	I'm concerned that what I share will reflect poorly of me as a resident and physician	○
○	I don't want to embarrass myself in front of my peers	○
○	I don't have enough time during the workday	○
○	I don't think it will help with addressing my wellness	○

Select the most and least significant factors that affected your participation in the residency-provided peer support group during intern year (Intern Forum) (group 7 of 7)

Most significant factor		Least significant factor
○	I don't like my classmates	○
○	I don't think it will help with addressing my wellness	○
○	I'm too tired	○
○	I don't want to embarrass myself in front of my peers	○

Block 3

Would you like to receive a $5 Amazon giftcard?

○ Yes

○ No

Powered by Qualtrics
